# “I am not really into the government telling me what I need to eat”: exploring dietary beliefs, knowledge, and practices among ethnically diverse communities in England

**DOI:** 10.1186/s12889-023-15689-6

**Published:** 2023-05-02

**Authors:** Abimbola S. Ojo, Lawrence A. Nnyanzi, Emma L. Giles, Louisa Ells, Sylvester R. Okeke, Kobi V. Ajayi, Obasanjo Afolabi Bolarinwa

**Affiliations:** 1 School of Health & Life Sciences, Teesside University, Middlesbrough, UK; 2 School of Health, Leeds Beckett University, Leeds, UK; 3 Centre for Social Research in Health, UNSW Sydney, Sydney, Australia; 4 Department of Health Behavior, School of Public Health, Texas A&M University, College Station, Texas United States; 5 Department of Public Health & Well-being, Faculty of Health & Social Care, University of Chester, Castle Drive, Chester, CH1 1SL UK; 6 Discipline of Public Health Medicine, School of Nursing and Public Health, University of KwaZulu-Natal, Durban, South Africa

**Keywords:** Dietary beliefs, Knowledge, Practice, Ethnically, Diverse, United Kingdom

## Abstract

**Background:**

Communities with diverse ethnicity in high-income countries are disproportionately affected by poor diet-related health outcomes. In England, the United Kingdom’s government’s healthy eating dietary resources are not well accepted and are underutilised among this population. Thus, this study explored perceptions, beliefs, knowledge, and practices around dietary intake among communities with African and South Asian ethnicity residing in Medway, England.

**Methods:**

This qualitative study generated data from 18 adults aged 18 and above using a semi-structured interview guide. These participants were sampled using purposive and convenience sampling strategies. All the interviews were conducted in English over the telephone, and responses were thematically analysed.

**Results:**

Six overarching themes were generated from the interview transcripts: eating patterns, social and cultural factors, food preferences and routines, accessibility and availability, health and healthy eating, and perceptions about the United Kingdom government’s healthy eating resources.

**Conclusion:**

The results of this study indicate that strategies to improve access to healthy foods are required to improve healthy dietary practices among the study population. Such strategies could help address this group’s structural and individual barriers to healthy dietary practices. In addition, developing a culturally responsive eating guide could also enhance the acceptability and utilisation of such resources among communities with ethnic diversity in England.

**Supplementary Information:**

The online version contains supplementary material available at 10.1186/s12889-023-15689-6.

## Introduction

Obesity is measured as a body mass index (BMI) of ≥ 30 kg/m^2,^ and its associated problems, such as cardiovascular diseases, type 2 diabetes mellitus, some types of cancer, and chronic diseases, present a significant global health challenge [1–3]. In the United Kingdom, one in every four adults has obesity, and over 10,780 hospital admissions are directly attributable to obesity [4, 5]. While multiple factors are associated with obesity, strong evidence links poor dietary patterns to the unacceptably high prevalence of obesity globally and in the United Kingdom. For example, the United Kingdom ranks third in volume sales of ultra-processed food per capita relative to other high-and-middle-income countries. Similarly, poor diets such as low intake of fruits and vegetables, whole grains, low-fat milk, nuts and seeds, fibres, and high intake of sugar, sodium, and processed meat are reportedly common in the United Kingdom [6, 7].

Although obesity is prevalent among all ethnic groups, disparities exist, with ethnic minorities bearing a disproportionate burden of disease [8]. Recent data suggest that racial/ethnic disparities in obesity begin from early childhood. In their research using the Millennium Cohort Study, Zilanawala and colleagues found that Black Caribbean and Black African children had an almost two-fold probability of obesity and being overweight after controlling for sociodemographic characteristics [9]. Similar findings have been reported in other studies in the United Kingdom [10–12]. Considering that there is a strong association between childhood adiposity and obesity in adulthood, it is not surprising that Black adults constitute a higher percentage (73.6%) of those living with overweight or obesity compared to their white British counterparts (63.3%) [13]. Additionally, one review study found that in addition to racial/ethnic differences, area-level deprivation, low educational attainment, and occupational social class increase the risk of obesity in United Kingdom adults [14], and minority populations commonly fall under these classifications [15].

Despite the clear link between poor dietary patterns and obesity and the consequent prevalence of obesity among minorities, their dietary beliefs, patterns, and knowledge remain largely unexplored. Previous research on minority dietary patterns suggests that minorities do not accept dietary health messages and interventions because they are not culturally responsive [16]. Furthermore, the lack/unavailability of traditional foods, taste preferences, and religious beliefs are barriers against healthy dietary practices among minoritised populations. Indeed, sociocultural environment and food beliefs and perceptions substantially shape minorities’ dietary behaviours [17, 18] due in part to migration and generational differences [16]. With the high influx of immigrants to Europe, it is important to prioritise understanding the dietary behaviours of minoritised groups to inform culturallyresponsive public health efforts in order to improve healthy eating habits among these groups.

To this end, this study elicited the insights of diverse minority ethnic groups in the United Kingdom on the factors influencing their dietary beliefs, patterns, and knowledge to fill literature gaps and help inform public health efforts. The study also aimed to improve understanding of the acceptability and utilisation of the current United Kingdom government’s dietary resources for the general population – Eatwell Guide – across diverse ethnic groups.

## Methods

### Design

This study was anchored on an interpretative orientation and used a qualitative approach to explore participants’ perceptions, beliefs, knowledge, and practices around dietary intake in Medway, England, United Kingdom.

### Participant recruitment

Purposive and convenience sampling strategies were used to recruit adult participants from communities with diverse ethnicity. Participants were recruited through the Health Action Charity organisation in Medway, England. Participants were invited to participate using the organisation’s mailing list. Invited participants who indicated interest were recruited and interview sessions were scheduled based on their convenience as regards date, time, and being interviewed over the telephone.

Health Action Charity Organisation is a charitable non-governmental organisation in Kent, United Kingdom, funded through fundraising and donations. The organisation provides information and raises awareness of the causes and consequences of health-related illness to members of African communities in Kent, Medway, United Kingdom.

### Data collection method – semi-structured interview

Semi-structured one-to-one telephone interviews were conducted between March and July 2019 (interview guide added as appendix I). The interviews lasted approximately 40 and 60 min and were conducted in English. Individual interviews provided platforms for participants to share their perceptions and practices in an environment where experiences were less likely to be biased by the experiences of other participants. The interview sessions explored participants’ eating patterns, including meal timing, consumption of traditional and “English” diets, healthy eating, and resources for healthy eating. All interview sessions were audio-recorded with participants’ consent. To allow for consistency in the interview process, one researcher with a shared identity from a minority-ethnic community as the participants performed the interviews.

### Data analysis

Interviews were transcribed verbatim, and the transcripts were analysed using reflexive thematic analysis [19–21]. This analytical framework was adopted to support a researcher’s interpretative, subjectivity, and deeper engagement with the study data in generating research knowledge [20–22]. In addition, previous studies involving data from ethnically diverse participants have used a thematic analytical framework partly to highlight differences and similarities in participants’ experiences [23, 24]. The analytical strategy used in this study combined inductive and deductive approaches. [20, 21, 25]. However, the themes generated from the interview transcripts were patterns of shared meanings identified in the data [20], not domain summaries. The analytical process involved reflexive reading of the interview transcripts multiple times for familiarisation and deeper understanding beyond semantic representations [20, 21]. The transcripts were then coded using NVivo. Theme generation involves searching, identifying, categorizing, naming, and refining the codes. It is important to stress that though two researchers (ASO & ELG) were involved in analysing the data, the essence was collaborative and aimed at enriching interpretations rather than for consensus [21, 22].

## Results

### Participant characteristics

The respondents interviewed for this study include eighteen (n = 18) participants with a mean age of 39, a standard deviation of 9, and an age range of 36. 78% of the respondents were first-generation migrants while 22% were second-generation migrants, 33% had below bachelor’s degree level while 67% had a bachelor’s degree. (Table 1).

**Table 1:** Participants’ characteristics

Variables	Counts (n = 18)
Age	
24–35	7
36–49	8
50 & above	3
**Migration status**	
First generation	14
Second generation	4
**Employment status**	
Employed	17
Unemployed	1
**Occupational Industry**	
Service	7
Health & Social Care	8
Financial	1
Clerical	1
Not applicable	1
**Educational attainment**	
Below bachelor’s degree	6
Bachelor’s degree	12

Table composed by the authors.

Age mean = 39; Age range = 36; Age standard deviation = 9.

## Generated themes

The qualitative analysis of the data gathered for this study yielded six main themes presented in Table 2 below. The interview guide is included in Appendix I.

**Table 2:** Overarching and sub-themes

Overarching Theme 1	Typical foods eaten by participants
Overarching Theme 2	Food preferences and routines
Overarching Theme 3	Social and cultural factors
Sub-themes	• The social and cultural meaning of food • Participants’ perception of their traditional food versus British food
Overarching Theme 4	Accessibility and availability
Sub-themes	• Availability of traditional food • Cost and time
Overarching Theme 5	Health and healthy eating
Sub-themes	• Nutrition and health • Perception of healthy eating
Overarching Theme 6	Perceptions around the United Kingdom government’s healthy eating resources
Sub-themes	• Barriers and facilitators to the usage of healthy eating resources • Culturally responsive healthy eating resources

### Theme 1: typical foods eaten by participants

Participants described their usual food as predominantly traditional, starchy, carbohydrate-based dishes comprising rice and other grains, with tomato sauce, meat, fish, vegetables, tubers, and special red oils, referred to as palm oil. The most popular dishes consumed were traditional vegetable soup, usually spicy and well-seasoned, and often eaten with rice or other starchy carbohydrates such as pounded yam or cassava (garri), and rice and curry soup. The main meal was usually consumed at lunch, described as heavy, with a slightly smaller portion of the same meal often consumed in the evening. Snacking between meals was common, with fruits and vegetables reportedly eaten by some of the participants. Mealtimes varied between individuals, depending on their work schedules. The majority of the participants reported having their breakfast between 08:00–10:00 am; the rest of the participants reported breakfast consumption between 06:00–07:30 am. Most participants said that they consumed their lunch between 12:00–2:00 pm, and for dinner, mostly later in the evening between 8:00–10:00 pm.

### Theme 2: food preferences and routines

The second overarching theme is related to food preferences and routines. Although participants stated that they preferred traditional food over British food, they stated that traditional food requires more time for preparation compared to British food. They perceived British dishes to be faster, more readily available, and more convenient. Some of the participants claimed that they struggle to combine their busy working lives with food preparation, and this prevents them from eating traditional food as often as they like:

*“Usually weekends, I cook traditional foods. My routine determines my food choices. If I have time, I will spend more time preparing my traditional foods. If I have a shorter time, I will make something like pasta, depending on the time. Work-life with family life altogether. Now it is harder.”* (SDN 05, 38-year-old female, Nigerian, second-generation immigrant).

Some participants also claimed that they eat British food while at work because it is more convenient and readily available:

*“British food is quick to pick up, readily available wherever you go, and it is convenient.”* (SDN 09, 35-year-old male, Nigerian, first-generation immigrant).

The frequency and times of eating traditional food and British food varied among the participants. Some participants claimed they eat traditional food every day, eating it for lunch or dinner, while others stated that they eat it at least once a week. The results also revealed that many participants eat British food for breakfast while traditional foods are reserved for lunch and dinner. They tend to take traditional food leftovers from dinner to work for lunch the next day:

*“If I’m working, I bring my traditional food as lunch from home. Which is what is left over from my dinner the previous day.”* (SDN 07, 37-year-old male, Nigerian, first-generation immigrant).

Generational differences in food preferences were recorded among the participants. Almost all participants who are first-generation immigrants (n = 14) indicated that they prefer traditional foods over British foods, and they eat their traditional food more frequently:

*“I love my African dishes. I take it regularly.”* (SDN 04, 60-year-old female, Ghanaian, first-generation immigrant).

In contrast, all participants who are second-generation immigrants (n = 4) claimed they prefer British foods and seldomly eat their traditional foods:

*“I don’t have it as much as English foods because of how heavy it is.”* (SDN 14, 25-year-old female, Ghanaian, second-generation immigrant).

Although participants stated that they eat fruit and vegetables every day, only four of them met the UK government recommendation of five fruit and vegetables a day:

*“Fruits and vegetables are my main food because of my medical condition. I take more than five fruits and vegetables a day.”* (SDN 06, 36-year-old male, Nigerian, second-generation immigrant).

### Theme 3: social and cultural factors

Within the overarching theme of social and cultural factors, two sub-themes were found: (1) the social and cultural meaning of food and (2) participants’ perceptions of their traditional food versus British food.

#### Sub-theme 1: social and cultural meaning of food

Participants, both first- and second-generation immigrants, acknowledged that the meaning of traditional foods, to them, went beyond the basic supply of health or energy and encompassed social and cultural meaning.

Many participants described traditional food as being familiar food with which they grew up, which was part of their family heritage.

Some of the participants described traditional food as a source of memories from their home country:

*“Most of the time when I don’t travel to Nigeria, but anytime I eat my traditional food here, it brings me back home. Especially how Nigeria is and how you grew up.”* (SDN 07, 37-year-old male, Nigerian, first-generation immigrant).

Some participants reported that they had food-related cultural and religious beliefs. Several participants admitted that they were not compliant with their religious injunctions when making food choices. They attributed their non-compliance to the poor availability of special foodstuffs, such as the scarcity of halal meat:

*“I eat some British foods. But because we don’t eat beef or pork, it has become difficult for us to choose.”* (SDN 15, 48-year-old female, Mauritian, first-generation immigrant).

#### Sub-theme 2: perception of traditional foods versus british food

Participants distinguished between their traditional food and British food. They took ownership of their traditional food by using personalised terms such as ‘my food’ or ‘food we eat’. In contrast, they refer to British food as ‘their food’ or ‘food they eat’. The importance of traditional food was expressed by some of the participants, who indicated that it is part of them and therefore it is very important.

They compared traditional food with British food in different terms and reported differences relating to taste, health benefits, satisfaction and methods of food preparation. Most of the participants (n = 14) described traditional foods as tastier, spicy and enjoyable; they thought that this was because traditional foods are garnished with different seasonings, which they claimed is lacking in British foods:

*“When I eat it, I enjoy the taste because that is the only time, I get to eat so much pepper. It is spicy; I like my food spicy.”* (SDN 01, 45-year-old male, Nigerian, first-generation immigrant).

The majority (n = 13) of participants referred to British food as less tasty, bland and not exciting. When they were asked to describe British food, some of the participants referred to it as boring. Indeed, one participant stated:

*“British food doesn’t taste great, in my opinion.”* (SDN 02, 24- year-old male, Nigerian, first-generation immigrant).

In contrast, some of the participants described British food as very tasty:

*“British foods are quite tasty; [it] all depends on what you choose to eat.”* (SDN12, 40-year-old female, South- African, first-generation immigrant).

### Theme 4: accessibility and availability of traditional foods

The third overarching theme relates to accessibility and availability of foods and is divided into two sub-themes: (1) availability of traditional food and (2) cost and time.

#### Sub-theme 1: availability of traditional foods

The availability of traditional foods in participants’ neighbourhoods and the quality of such foods were discussed. The findings in this study do not indicate a problem with the availability of traditional foods in local shops; indeed, many of the participants expressed their relief that many food shops in their neighbourhoods sell traditional foods. Consequently, they did not have to travel far and believed that traditional foods are available and can be accessed easily:

*“The foods are available in Medway; you can also travel to London to buy them, they are available.”* (SDN 10, 50-year-old female, Zimbabwean, first-generation immigrant).

That said, some participants still preferred to travel to London to buy their foodstuffs because of the belief that such items are fresher and are of higher quality, including a variety of special ingredients, compared to those available in local shops:

*“We tend to go to London to get our foods. Hamm in terms of quality; I just prefer to buy in London. The one they sell in London is fresher than the one they sell around [here].”* (SDN 11, 30-year-old male, Nigerian, first-generation immigrant).

#### Sub-theme 2: cost and time

Participants’ perceptions of the influence of cost on their food choices differed from individual to individual. However, it was observed that participants’ socioeconomic status (SES) and age played a factor in how each participant perceived the impact of cost on their food choices. When participants were asked about the factors that influence their food choices, although some did not view cost as a prerequisite, they believed that traditional foods are affordable, especially for the working class. Those with the opinion that the cost of food did not influence their food choice were found to be participants with medium/high SES or younger participants with lesser financial commitments. For instance, one participant, who is a professional nurse, stated:

*“If you are working, you can buy any food in this country. It is not that expensive in the Asian shop; you can buy three plantains for £1.20, and that is not too much, and it can be served between three people in the house.”* (SDN 04, 60-year-old, female, Ghanaian, first-generation immigrant).

In contrast, the cost of food had a great influence on food choices for some participants with low SES. They stressed that the first thing they consider before buying any food is whether they can afford it. If the food is too expensive, they will instead look for bargains or alternatives. This experience was shared by one of the unemployed participants:

*“… I look at the price; to be honest, I don’t look at the ingredients that much.”* (SDN 08, 34-year-old female, Zimbabwean, first-generation immigrant).

Moreover, time was also a crucial factor in participants’ food choices. Many participants highlighted that their work schedule and busy lifestyles sometimes prevent them from eating their preferred food. Some participants indicated that they have no choice but to regularly eat ‘takeaway’ food because of their busy schedules:

“*We buy takeaway because both of us work; it depends on when I and my wife get home. If it is in the evening because both of us are working and we have to feed the children, we might grab something. We will buy fish and chips on our way home or quick takeaway like Madonna’s, just for convenience.”* (SDN 03, 45-year-old male, Turkish, second-generation immigrant).

### Theme 5: health and healthy eating

Another overarching theme identified in the interview transcript was health and healthy eating, which was categorized into two subthemes: (1) nutrition and health and (2) perception of healthy eating.

#### Sub-theme 1: nutrition and health

Participants reported that healthy eating was important because it benefitted their health. Therefore, they tried to substitute unhealthy foods with healthy options by eating low-calorie foods instead of traditional starchy foods. This was to prevent non-communicable diseases, such as diabetes:

*“We are trying to avoid white potatoes, the sweet potatoes because of these diabetes things happening, so we tend to look at what is recommended as healthier every time. We do eat them but not as much. So, it’s brown rice, brown bread, even the bread we have cut down bread to be sometimes we don’t eat bread in the house for two good months.”* (SDN 10, 50-year-old female, Zimbabwean, second-generation immigrant).

The participants also discussed the perceived health benefits of foods. Some participants believed that certain traditional foods are medicinal:

*“Pepper soup clears my system; my body feels good, and all my organs are working well. If I am feeling cold, I eat pepper soup.”* (SDN01, 45-year-old male, Nigerian, first-generation immigrant).

However, some participants rightly noted that healthy eating alone, without being physically active, is not enough for good health.

*“But if you eat a healthy diet and not too much and you do exercise you will be healthy.”* (SDN 07, 37-year-old male, Nigerian, first-generation immigrant).

#### Sub-theme 2: perception of healthy eating

Many participants perceived themselves to have good knowledge about healthy eating. All participants agreed that healthy eating involves a balanced diet containing the correct proportion of nutrients. Many participants agreed that eating in moderation is healthy, implying that one should not eat too much at once. Some participants mentioned that consuming less fat is healthy. Almost all participants mentioned that substituting red meat with fish or chicken and not eating too many eggs was healthy eating:

*“Vegetables, the greens, broccoli, peas. In terms of going deeper, it means [a] balance[d] diet, having a variety of different vitamins within your dietary intake, not having full meal but having an adequate meal at regular intervals.”* (SDN 05, 38-year-old female, Nigerian, second-generation immigrant).

In addition to fruit and vegetables, among common healthy foodstuffs, the participants mentioned some traditional starchy foods. Unprocessed and fresh foods were also classified as healthy food:

*“Fruit and vegetables because I like my salad as well. They are the healthiest foods that I consume, and my home-cooked stew, whether rice or pasta, my pounded yam and okra or spinach soup. Those are the type of foods that I believe is healthy. As long as they are not processed and freshly made, I believe it is healthy.”* (SDN 07, 37-year-old male, Nigerian, first-generation immigrant).

### Theme 6: perceptions of healthy eating resources

Within the overarching theme of perceptions of healthy eating resources, two sub-themes were identified: (1) barriers and facilitators to the use of healthy eating resources and (2) culturally responsive healthy eating resources.

#### Sub-theme 1: barriers and facilitators to the use of healthy eating resources

Almost all participants were aware of the daily five fruits and vegetables recommendation. Participants were also aware of health-related information on food packaging, but only a few participants, who were healthcare workers, claimed to have heard of the Eatwell Guide.

With regards to participants’ awareness of healthy eating resources, some participants stated that they do not use the resources:

*“I must admit, I don’t use any of them. I don’t even look at colours or calories when I buy food … I have never looked at the guides - I … never have.”* (SDN 12, 40-year-old female, South-African, first-generation immigrant).

Barriers to using the UK’s healthy eating resources included a lack of nutritional knowledge and a perception that they are not well marketed to create widespread awareness. Some of the participants reported that they would use healthy eating resources if there was greater publicity and awareness:

*“I hear about it, like now it will get me to think about it and the implication on my health. It will affect me a little while before it goes away from me.”* (SDN 05, 38-year-old female, Nigerian, second-generation immigrant).

Other barriers to the use of healthy eating resources mentioned by participants included misconceptions of healthy eating and poor attitudes toward healthy eating. Even if individuals have good knowledge, some participants still felt this would not make any difference; people will continue to eat their usual foods. One participant expressed dissatisfaction with the government trying to impose healthy eating on people:

*“I am not really into the government telling me what I need to eat. I eat what I think is good for me and what I enjoy eating. People don’t bother. They just eat.”* (SDN 07, 37-year-old male, Nigerian, first-generation immigrant).

#### Sub-theme 2: culturally responsive healthy eating resources

The participants discussed the appropriateness of government healthy eating resources in terms of accessibility of accessing information, how the resources have failed to meet their requirements, and the difficulties that participants encountered in trying to adapt the information to traditional foods. Some participants believed that the United Kingdom government only targets the white majority group with healthy eating resources, and examples of traditional multi-ethnic foods are not included in the healthy eating resources:

*“I think they have targeted the majority of people. The percentage of the black people is not as much when compared with the white, maybe that is why they use the foods that majority are familiar with.”* (SDN 11, 30-year-old male, Nigerian, first-generation immigrant).

It is likely that the government’s source of healthy eating information may not be appropriate or accessible to communities with diverse ethnicity:

*“I know the public health [Public Health England] is responsible for all those things. The council Public Health will probably help the department to do all those things, but we don’t normally go to all those places.”* (SDN 10, 50-year old female, Zimbabwean, first-generation immigrant).

Other participants believed that some communities with minority ethnicity would not normally seek information through a government’s website. Some participants shared that most individuals from communities with minority ethnicity may have a poor attitude to checking government information from official websites. According to perspectives shared at the interviews, people from communities with diverse ethnicity in the United Kingdom would rather seek information from social media or rely on their social or religious groups:

*“I will rather seek information through social media or magazines on healthy food. Then I compare it with my local foods; sometimes they set the alertness of dangers of most of the foods like palm kernel.”* (SDN 04, 60-year-old female, Ghanaian, first-generation immigrant).

## Discussion

This study explored dietary practices and its associated factors among immigrants from ethnically diverse communities in the South of England. The study’s results suggest that participants prioritise social and cultural values over health and nutrients when making food choices. In line with previous evidence [26, 27], we found that immigrants (especially first-generation immigrants) in England were more disposed to their traditional cuisine. In addition, participants attached great importance to preserving and passing their culinary traditions and dietary norms to future generations. [28]. Furthermore, our study revealed participants encountered structural barriers, constraining their ability to consume traditional meals and/or engage in healthy dietary practices, similar to studies in high-income countries [29].

Contrary to previous studies [28–30], participants’ nutritional knowledge mitigated barriers to healthy dietary practices. Participants in this study demonstrated awareness and adequate knowledge of healthy eating and exercise for health, and such knowledge may not translate to healthy dietary practices for some participants because of structural and individual-level barriers [29].

Migrant populations in high-income countries who come from low- and middle-income settings are known to be at a higher risk of socio-economic vulnerabilities. These vulnerabilities may also involve being in occupations that impact good work-life balance [29]. Consequently, this population may find it difficult to have adequate time to plan and prepare meals based on preferences and/or health benefits [31]. As the results of this study clearly indicate, bustling to and from work and other activities required to sustain livelihood may impact nutritional patterns and dietary practices [29, 31]. Therefore, it is not uncommon that some participants reported relying on commercially available fast foods, which may not meet recommended nutritional needs. Even though healthy choices can be made in picking commercially available fast food, the time needed for planning and enjoying a wholesome meal may not be available [29]. Thus, this study’s results align with previous findings indicating busy schedules as a barrier to healthy dietary practices among minority groups in high-income settings [29, 32].

Similarly, and partly related to socioeconomic vulnerabilities, the economic cost of foods may constrain the dietary practices of this population. In line with previous studies [33–35], this study’s results re-echo the extent to which cost impacts healthy nutritional practices among migrant populations in high-income settings [35]. Though some participants fully understood that some food products are healthy and nutritious, the cost of purchasing these items became a barrier to doing so. This is especially the case for participants who were experiencing a higher level of socio-economic vulnerability and a lower socio-economic status [33]. This sub-population reported affordability as the most important factor in shaping their food choices [29]. Thus, beyond tokenistic interventions, such as providing nutritious food to this group, addressing socio-economic vulnerability through economic empowerment could be a strategic pathway to empowering this population to practice healthy nutritional behaviours [34]. Economic empowerment for enhanced purchasing power is preferred to unsustainable nutritional palliatives [35].

Furthermore, the results of this study indicate the unmet needs of communities with diverse ethnicity regarding culturally responsive and inclusive dietary resources. Though some participants were aware of the United Kingdom government’s healthy eating initiatives, the level of acceptability of the resources was low [36]. Interestingly, some participants perceived the United Kingdom’s eating guidelines as not applicable to them because the guidelines do not include their traditional foods [37, 38]. Consequently, these healthy eating guidelines are being perceived as applicable only to the white British, whose cuisines are mentioned in the resources. This finding aligns with results from a systematic review of 20 studies across six countries, indicating the non-acceptability of inequitable health-related tools and resources among communities with diverse ethnicity [32, 38]. This non-acceptance, especially in a multicultural society, could be because these resources fail to account for the diverse needs and experiences of minority populations.

### Strengths and limitations

To our knowledge, this study is the first to qualitatively explore dietary perceptions, beliefs, knowledge, and practices among communities with diverse ethnicity in Medway, England. Besides exploring dietary practices, this study also appears to provide a foundational knowledge of the acceptability and use of the United Kingdom government’s dietary resources among communities of diverse ethnicity. Therefore, the results of this study provide empirical insights that could inform the development of culturally responsive dietary resources to promote healthy eating among communities with diverse ethnicity in the United Kingdom. In addition, participants were interviewed by a member of the study population. This membership facilitated the collection and interpretation of rich and robust data as it helped to bridge cultural barriers that could impact qualitative data collection.

However, the results of this study need to be interpreted within caution. The results of this study may not be generalised, being a qualitative study, as data was generated from a small sample in Medway. Replicating this study in various settings in the United Kingdom could provide a broader picture of the dietary patterns of ethnically diverse communities across the United Kingdom. Notably, all participants were fluent in the use of English. It is not unlikely that the language barrier may have impacted the willingness to participate. Thereby, the voices of non-English speaking members of the study population may not be captured in the results of this study. Also, we did not explore how sociodemographic characteristics (e.g., annual household income, educational attainment, employment, profession, household structure, [how many adults and children live in the household], food security, weight status) shape eating patterns. We suggest that future studies examine how these characteristics shape dietary and eating patterns. Moreover, the recruitment of participants was based on convenience sampling, which is not representative of entire communities of immigrants. In addition, the Health Action Charity Organisation was the only gatekeeper for the recruitment of participants in this study, which may have affected the ethnic diversity of participants.

Furthermore, as participants were members of the Health Action Charity Organisation in Medway, England, many were healthcare workers, which may have biased the sample in such a way that a higher proportion of the participants had a higher level of health literacy than the wider population. Lastly, this study was conducted in 2018; advances in public health efforts might have occurred within this time span, which may invalidate some of our findings. However, given the prevalence of obesity in the United Kingdom over time, it is likely that our results still reflect current circumstances.

### Implications for research policy and practice

Despite the limitations of this study, its results constitute an important contribution with highly relevant implications for research, policy and practice. In terms of scholarship, the results of this study provide an empirical foundation for future researchers to further examine population-specific strategies to improve dietary practices among ethnically diverse communities in the UK and other high-income countries. This is important, considering that these high-income countries are becoming increasingly multicultural and ethnic minority groups continue to be disproportionately affected by nutrition-related health issues [8]. As regards policy, this study indicates a need for more inclusive health-related policies to drive governmental responses to real and envisaged public health issues. Finally, in terms of practice, this study supports evidence [39] for adopting co-design approaches to developing health-related resources, especially in a highly multicultural environment.

Co-designed dietary guidelines could address minoritizing population groups who may see non-inclusive guidelines as being thrust on them. This is because a co-designed approach would ensure the guidelines are comprehensive, inclusive and reflect cultural diversity. This way, every population group has a sense of belonging, viewing such inclusive dietary guidelines as tools that apply to everyone and not just to a particular group. Besides ensuring diversity in the public health workforce, it is also imperative to enlist the active participation of diverse socio and ethnocultural communities in planning, implementing and evaluating health promotion tools such as dietary guidelines.

Moreover, the results of this study have implications for cultural maintenance in relation to foods and dietary practices of second-generation migrants and young people who are disproportionately impacted by poor dietary outcomes [9]. Our results clearly showed that second-generation migrants might be losing touch with their traditional meals and diets. In a multicultural society and within global mobility, it is important to preserve material and non-material cultures and practices that are safe and health-supportive. Against this background, our study results have implications for promoting healthy traditional diets and practices among young people to improve their nutritional health and preserve their cultural heritage and identity. To this end, relevant health and educational policies and targeted health promotion messages could be developed, implemented, and periodically evaluated using the participatory approach. The social media platform could serve as a veritable tool for targeted health promotion outreaches, using group pages of target populations. Reaching racial and ethnically diverse groups with inclusive dietary guidelines could help improve their nutritional practices, thereby saving funds, time and efforts expended in managing nutrition-related health problems.

## Conclusion

Based on the results of this study, it is clear that structural and individual-level constraints impact the dietary practices of immigrant communities in Medway, England. In addition, non-inclusive public health tools and resources constrain the extent to which this population engages with and utilises the recommendations in such tools and resources. Therefore, a more inclusive and equitable approach to developing and deploying dietary guidelines must be adopted. In addition, given other structural constraints faced by communities with diverse ethnicity, strategies to improve the availability and accessibility to healthy foods are essential to facilitate the adoption of healthy dietary practices among this population.

## Electronic supplementary material

Below is the link to the electronic supplementary material.

Supplementary Material 1

## Data Availability

All data and materials are included in the manuscript.
