# Supplementary material for: “I am not really into the government telling me what I need to eat”: exploring dietary beliefs, knowledge, and practices among ethnically diverse communities in England
Source: BMC Public Health. 2023 May 2;23:800. doi: 10.1186/s12889-023-15689-6 (PMC10152749; doi:10.1186/s12889-023-15689-6)
Supplement: Supplementary file 1 — Supplementary Material 1 [file 12889_2023_15689_MOESM1_ESM.docx]

**Appendix I: Interview Guide**

**Exploring the perceptions, beliefs, knowledge, and practices of healthy eating among Black, Asian and Minorities Ethnic groups in Medway, England.**

**Aims and Objectives**

The aim of this study to explore the perceptions, beliefs, knowledge, and practices around dietary intake among BAME adults (18 years and over) in Medway.

The objectives are:

- To explore perceptions, beliefs, knowledge, and practices around dietary intake among Black, Asian and Minorities Ethnic (BAME) groups.
- To assess the BAME awareness, perceptions, barriers and use of the current UK government dietary resources.
- To use the findings from this study to develop a list of recommendations to inform the development of culturally responsive resources to promote healthy eating and prevent obesity among the BAME in the UK.

**The aim of this study is to**

**Introduction**

- Introduce self to the participants**.**
- Introduce the study - who is it for, what is it all about.
- Talk through the key points

Purpose of this interview

Duration of the interview

-Voluntary participation and right to withdraw.

- Confidentiality, anonymization, recording

- Check for any questions participants have

**Eating Patterns and Mealtimes.**

- How will you describe your typical food for breakfast?
- How will you describe your typical food for lunch?
- How will you describe your typical food for dinner?
- Can you mention types of food you eat for breakfast?
- Can you mention types of food you eat for lunch?
- Can you mention types of food you eat for dinner?
- Do you snack between your meals?
- If yes, what are the types of snacks you normally eat between meals?
- Are fruits and vegetables part of your daily meal?
- What type of fruits and vegetables do you eat regularly?
- How many fruits and vegetables do you eat each day?
- Do you often take home cooked food?
- How do you normally prepare your food?

**Traditional Diets**

- What do you understand by traditional foods?
- How would you describe your traditional foods?
- What do traditional foods mean to you?
- How often do you eat your traditional foods?
- How do you feel about eating your traditional foods?
- How important are your traditional foods to you?
- Do you have any cultural or religious belief that influences which foods you eat?
- If yes, could you talk me through these?
- How easy is it for you to buy your traditional foods?
- What do you normally consider before you buy your food?

**United Kingdom Diets**

- What do you understand by English foods?
- How would you describe English foods?
- Do you consume English foods?
- If yes, what time of the day do you normally eat English foods?
- Could you describe types of English foods you normally eat?
- Why do you eat particular types of English foods?
- What influences your food choices?

**Healthy Eating**

- What comes to your mind when you hear the term ‘healthy food’?
- Could you mention some healthy foods you normally eat?
- What are the factors that may affect what foods you eat?
- Do you believe that you have access to healthy food?

**Health Eating Resources**

- Are you aware of any government healthy eating resources?
- Prompt: Eatwell Guide, food pack labels and five fruits and vegetables recommendations?
- Have you used the Eatwell Guide before?
- Do you normally use front-of-pack food labels to choose your foods?
- Where do you go to in order to get information on healthy foods?
- Do you normally look at food labels before you buy your food?
- If yes, what information on the labels do you look at and why?
- What do you think are the barriers for not using government resources to inform healthy eating behaviours?
- What are the reasons why you would use government resources to inform healthy eating behaviours?
- How do you think these resources are suitable for the BME groups?
- What would you like to change to make these resources suitable for BME use?
- Do you have any other comments on the healthy eating resources?

**At the end of this telephone interview**

- Thank participant for taking part in an interview
- Provide reassurance about confidentiality
- Check if the participant want to be informed of findings
- Check whether the participants have any questions
